# Association between changes in harm perceptions and e-cigarette use among current tobacco smokers in England: a time series analysis

**DOI:** 10.1186/s12916-020-01565-2

**Published:** 2020-05-06

**Authors:** Olga Perski, Emma Beard, Jamie Brown

**Affiliations:** Department of Behavioural Science and Health, University College London, 1-19 Torrington Place, London, WC1E 6BT UK

**Keywords:** E-cigarettes, Harm perceptions, Tobacco smoking, Time series analysis, ARIMAX

## Abstract

**Background:**

There is a decreasing trend in the proportion of individuals who perceive e-cigarettes to be less harmful than conventional cigarettes across the UK, Europe and the US. It is important to assess whether this may influence the use of e-cigarettes. We aimed to estimate, using a time series approach, whether changes in harm perceptions among current tobacco smokers have been associated with changes in the prevalence of e-cigarette use in England, with and without stratification by age, sex and social grade.

**Methods:**

Respondents were from the Smoking Toolkit Study, which involves monthly cross-sectional household surveys of individuals aged 16+ years in England. Data were aggregated monthly on ~ 300 current tobacco smokers between 2014 and 2019. The outcome variable was the prevalence of e-cigarette use. The explanatory variable was the proportion of smokers who endorsed the belief that e-cigarettes are less harmful than combustible cigarettes. Covariates were cigarette (vs. non-cigarette combustible) current smoking prevalence, past-year quit attempt prevalence and national smoking mass media expenditure. Unadjusted and adjusted autoregressive integrated moving average with exogeneous variables (ARIMAX) models were fitted.

**Results:**

For every 1% decrease in the mean prevalence of current tobacco smokers who endorsed the belief that e-cigarettes are less harmful than combustible cigarettes, the mean prevalence of e-cigarette use decreased by 0.48% (*β*_adj_ = 0.48, 95% CI = 0.25–0.71, *p* < .001). Marginal age and sex differences were observed, whereby significant associations were observed in older (but not in young) adults and in men (but not in women). No differences by social grade were detected.

**Conclusions:**

Between 2014 and 2019 in England, at the population level, monthly changes in the prevalence of accurate harm perceptions among current tobacco smokers were strongly associated with changes in e-cigarette use.

## Introduction

Cigarette smoking is one of the leading causes of premature morbidity and mortality; each year, 8 million people worldwide die of smoking-related disease [1]. Electronic cigarettes (e-cigarettes) are battery-powered devices that deliver nicotine without burning tobacco and are less harmful than conventional cigarettes [2–5]. E-cigarettes are rising in popularity in high- and middle-income countries, including the United Kingdom (UK), Europe and the United States (US), and their use is positively associated with the success of quit attempts at the population level [6, 7]. A growing body of research has studied the prevalence and correlates of e-cigarette harm perceptions, with a focus on the perceived relative harm of e-cigarettes compared with conventional, combustible cigarettes. These perceptions are associated with the trial and use of e-cigarettes cross-sectionally [8–12] and prospectively [13].

The proportion of adults who perceive e-cigarettes to be less harmful than conventional cigarettes range from 21 to 85%; estimates vary by country and year [12, 14–23]. Harm perceptions are influenced by media depictions of e-cigarettes, increased use and marketing [11, 20, 24]. It is assumed that negative media portrayals of e-cigarettes reduce accurate harm perceptions. Consistent with this concern, during a period of numerous e-cigarette news stories focusing on absolute risks, longitudinal surveys of nationally representative samples report a decreasing trend in the proportion of individuals who perceive e-cigarettes to be less harmful than conventional cigarettes [17, 20, 21, 25–28]. However, to our knowledge, no study to date has used a time series approach to estimate whether changes in harm perceptions are associated with changes in the prevalence of the use of e-cigarettes at the population level. We used data from the English Smoking Toolkit Study to address this question; the application of time series analysis allowed us to describe the association after removing potential trend and seasonality components.

Previous research has identified individual-level predictors of harm perceptions and has found significant differences by age, sex and social grade: younger age [11, 16, 20, 25], male sex [11, 20, 21, 27, 29] and higher income/education [16, 20, 21, 25] are associated with greater odds of endorsing the statement that e-cigarettes are less harmful than conventional cigarettes. However, some studies have found that older age [13, 14, 27], female sex [16] and lower income/education [27] are associated with greater odds of believing that e-cigarettes are less harmful. It is, however, unclear whether these individual-level predictors remain important at the population level. We therefore stratified our analyses by age, sex and social grade.

Specifically, this study addressed the following research questions:

1. At the population level, is there an association between changes in the monthly prevalence of tobacco smokers with accurate e-cigarette harm perceptions and e-cigarette use?
2. Does the association differ by age, sex or social grade?

## Methods

### Study design and setting

STROBE guidelines were followed throughout [30]. The study protocol and analysis plan were pre-registered on the Open Science Framework (https://osf.io/ze5hf). The study is part of the ongoing Smoking Toolkit Study (STS) which involves monthly, face-to-face, computer-assisted household surveys of adults aged 16+ in England [31]. The sample is a hybrid of a random probability and quota sample, which results in a sample that is representative of the adult population of past-year smokers in England [31, 32]. Interviews are held with one household member. Interviewers travel to selected output areas and perform computer-assisted interviews with one household member aged 16+ years until quotas based on factors influencing the probability of being at home (i.e. working status, age and gender) are fulfilled. In this hybrid form of random probability and quota sampling (which is considered superior to conventional quota sampling), the choice of households to approach is limited by the random allocation of small output areas. Rather than being sent to specific households in advance, interviewers can choose which households within these areas are most likely to fulfil their quotas. Unlike random probability sampling, where interviewers have no choice as to the households sampled and can record responses at each address, it is not appropriate to record response rates in the Smoking Toolkit Study. Comparisons with national survey and sales data indicate that key demographic variables, smoking prevalence and cigarette consumption are nationally representative [31, 32]. Informed consent is obtained prior to each interview. Ethical approval was granted by UCL’s Research Ethics Committee (2808/005).

### Study population

Data included in the present study were collected from respondents surveyed between November 2014 (when the survey item about harm perceptions was first included in the STS) and May 2019 (the latest wave of data available). Respondents were aged 16+ years at the time of the survey and were included in the analyses if they (i) were current tobacco smokers (e.g. manufactured or hand-rolled cigarettes, pipe, cigars or shisha) at the time of the survey; (ii) had complete data on age, sex and social grade; (iii) had complete data on past-year quit attempts; and (iv) had complete data on e-cigarette harm perceptions. Individual-level data were aggregated to produce population-level estimates for the variables of interest.

### Measures

The outcome variable was the prevalence of e-cigarette use among current tobacco smokers. Respondents who report that they are smoking tobacco daily or occasionally were asked to answer the following questions by selecting or not selecting options from a list of nicotine products (including e-cigarettes):

1. ‘Which, if any, of the following are you currently using to help you cut down the amount you smoke?’
2. ‘Do you regularly use any of the following in situations when you are not allowed to smoke?’
3. ‘Can I check, are you using any of the following either to help you stop smoking, to help you cut down, or for any other reason at all?’

The prevalence of e-cigarette use among smokers was obtained for each survey wave by counting the number of respondents who endorsed the use of e-cigarettes in response to any of the three questions above, divided by the total number of current tobacco smokers.

The explanatory variable was e-cigarette harm perceptions among smokers, assessed by asking: ‘Compared to regular cigarettes, do you think electronic cigarettes are more harmful, less harmful, or equally harmful to health?’ This item was coded 1 for respondents who selected the option ‘less harmful’ and 0 otherwise (i.e. ‘more harmful’, ‘equally harmful’, ‘do not know’).

Covariates included cigarette (vs. non-cigarette combustible) smoking prevalence, prevalence of past-year quit attempts (an indicator of motivation to stop and a potent individual-level predictor of e-cigarette use [33, 34]) and national smoking mass media expenditure, with raw data (in millions) on quarterly expenditure in British pounds, not adjusted for inflation (i.e. nominal as opposed to real expenditure), obtained from Public Health England in June 2019.

We also stratified the analyses by age (16–24 years, 25–64 years, 65+ years), sex (male vs. female) and social grade (C2DE vs. ABC1), assessed by the British National Readership Survey’s Social Grade Classification Tool [35]. This occupational measure of social grade is a valid indicator of socioeconomic status that is widely used in research in the UK population. It has been identified as particularly relevant in the context of tobacco smoking [36]. The social grades A, B, C1, C2, D and E are frequently combined into two categories: ABC1 and C2DE. Here, researchers often interpret ABC1 to represent the middle class and C2DE to represent the working class. This grouping also ensured that sample sizes were adequate to give accurate aggregated estimates for the stratified analyses.

### Data analysis

The analyses were conducted in R v.3.5.1 using the *arimax* function in the *TSA* package. Data were weighted using the rim (marginal) technique [37] to match English census data on age, sex and social grade. A series of unplanned descriptive analyses were conducted as a result of the review process. First, we assessed whether respondents with missing data differed systematically from those with complete data on the exposure and outcome variables of interest. Second, we plotted the proportion of respondents endorsing the belief that e-cigarettes are ‘more harmful’, ‘equally harmful’, ‘less harmful’ and ‘do not know’ over the study period. Third, we conducted two descriptive linear trend analyses to assess whether the prevalence of e-cigarette use and/or the proportion of respondents who endorsed the belief that e-cigarettes are less harmful than combustible cigarettes declined significantly over the course of the study period.

Autoregressive integrated moving average with exogenous variables (ARIMAX) models were fitted to estimate the association between changes in harm perceptions and changes in the use of e-cigarettes among current tobacco smokers, with and without stratification by age, sex and social grade. As this was a time series analysis using data aggregated at the population level, it was not possible to test for interactions as one can do with individual-level data [38]. We decided a priori to stratify the analyses to describe whether the magnitude of the association was similar across demographic groups rather than assessing if the effect would differ across groups as we would in a moderation analysis.

We also conducted a sensitivity analysis (SA) without stratification on the basis that respondents who select the option ‘do not know’ to the survey item asking about e-cigarette harm perceptions may be usefully categorised as being open to the possibility of using e-cigarettes (as opposed to no interest in use). In the SA, we hence collapsed respondents who selected the options ‘less harmful’ and ‘do not know’.

We followed a standard ARIMAX modelling approach [39], detailed in Additional file 1. The output series was first differenced and log transformed to stabilise the variance [40, 41]. To facilitate comparison across the main and stratified analyses, the series were standardised by subtracting the mean, dividing by the standard deviation and adding a constant of 10 (to prevent negative values). Plots of the autocorrelation and partial autocorrelation functions were examined to identify plausible values for the autoregressive (AR) and moving average (MA) terms for the baseline model. The cross-correlation functions were then assessed with pre-whitened data to identify the most appropriate transfer functions (i.e. the manner in which past values of the input time series predict future values of the output time series) for the explanatory variables [42]. ARIMAX models assume that there is weak exogeneity between the input and output time series (meaning that the output series can depend on lagged values of the input series but not the other way around) [43]. To assess this, we used the Granger Causality Test, which regresses each time series onto lagged values of itself and of the other time series [43]. Different models with plausible AR and MA terms were then compared with the baseline model using the Akaike Information Criterion (AIC), with smaller values indicating better model fit. Coefficients were reported for the best fitting models alongside the pseudo *R*^2^, calculated here as the squared correlation between the fitted and the actual values, with values ranging between 0 (poor fit) and 1 (perfect fit). As differencing was used to render the time series stationary, the model coefficients can be interpreted as the percentage change in the mean of the output series (i.e. e-cigarette use) as a result of a 1% increase or decrease in the mean of the input series (i.e. e-cigarette harm perceptions).

### Sample size

There are no clear sample size recommendations for ARIMAX models, but as these models are specified in a similar way to ARIMA models, the same criteria are likely to apply. Some suggest at least 50 observations, although it has been argued that these models are suitable for shorter time series as long as there are more observations than model parameters. A total of 55 observations were available.

## Results

**Fig. 1:**
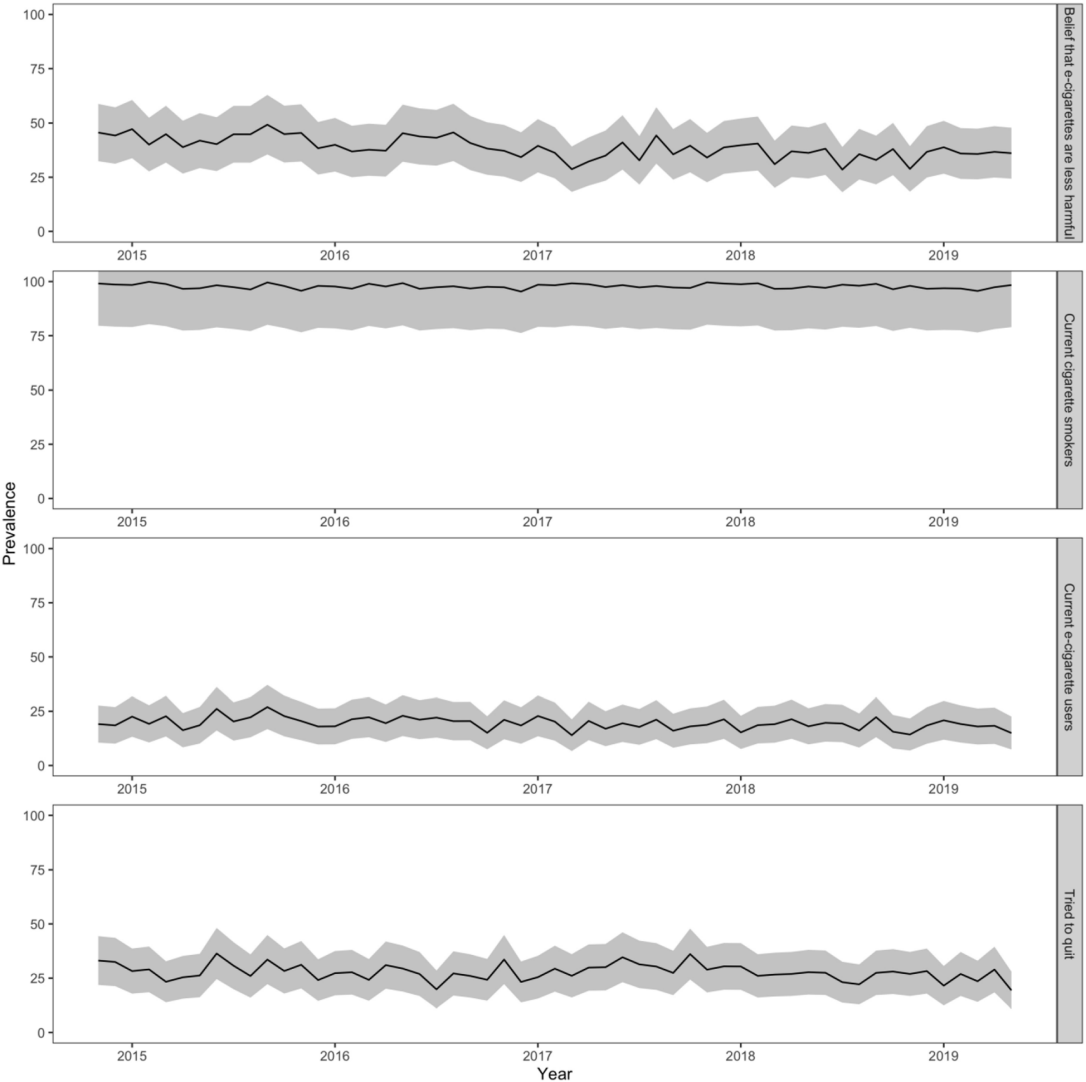
Monthly prevalence of current tobacco smokers endorsing the belief that e-cigarettes are less harmful than combustible cigarettes, cigarette smokers, e-cigarette users and past-year quit attempts and associated 95% confidence intervals

A total of 16,567 current tobacco smokers were surveyed. Of these eligible respondents, 79 (0.5%) had missing data on age and 487 (2.9%) had missing data on past-year quit attempts. A total of 16,009 (96.6%) respondents had complete data on all variables of interest. Those with missing data were significantly less likely to use e-cigarettes (14.3% vs. 19.3%; *p* < .01) but not to endorse the belief that e-cigarettes are less harmful than combustible cigarettes (34.6% vs. 38.5%; *p* = .06). The prevalence of e-cigarette use declined significantly over the course of the study period (*B* = − 0.07, *p* < .01). The prevalence changed from 19.1% at the start of the study period to 15.0% in the last month of the study period (*M* = 19.5%, SD = 2.7%). The proportion of respondents who endorsed the belief that e-cigarettes are less harmful than combustible cigarettes also declined significantly (*B* = − 0.19, *p* < .001) from 45.6% at the start of the study period to 36.1% in the last month of the study period (*M* = 38.8%, SD = 4.8%; see Additional file 2: Fig. S1 for descriptive plots of smokers’ harm perceptions using the original 4-level coding). There was an increase followed by a decline in the proportion of respondents who reported making a quit attempt in the past year. The prevalence changed from 33.1% at the start of the study period to 19.4% in the last month of the study period (*M* = 27.8%, SD = 3.7%). The proportion of current cigarette (vs. non-cigarette combustible) smokers remained stable throughout the study period (*M* = 97.8%, SD = 1.1%) (Fig. 1).

### Association between harm perceptions and e-cigarette use in the total sample

In unadjusted and adjusted analyses, at the population level, changes in the proportion of tobacco smokers who endorsed the belief that e-cigarettes are less harmful than combustible cigarettes were significantly associated with changes in the proportion who use e-cigarettes (see Table 1). For every 1% decrease in the mean prevalence of tobacco smokers who endorsed this belief, the mean prevalence of e-cigarette use decreased by 0.48%. Additional file 2: Figure S5-S9 shows the fitted compared with the actual values of the output time series.

**Table 1:** Estimated percentage point change in the mean prevalence of e-cigarette use among tobacco smokers who endorse the belief that e-cigarettes are less harmful than combustible cigarettes during the study period (2014–2019), based on autoregressive integrated moving average with exogenous input (ARIMAX) models

	Unadjusted (95% CI, *p* value)	Adjusted (95% CI, *p* value)
**Percentage change per 1% change in the mean prevalence of the exposure**		
Use of e-cigarettes	0.52 (0.30–0.75), < 0.001	0.48 (0.25–0.71), < 0.001
Current cigarette smokers	–	0.08 (− 0.14–0.31), 0.48
Tried to quit in the past year	–	0.12 (− 0.11–0.36), 0.31
National mass media expenditure in millions (£)	–	0.10 (− 0.14–0.34), 0.42
**Best fitting model**		
ARIMAX (*p*,*d*,*q*)(*P*,*D*,*Q*)	(0,1,1)(0,0,0)	(0,1,1)(0,0,0)
Non-seasonal (*p* value)		
Autoregressive (AR) term	–	–
Moving average (MA) term	< 0.001	< 0.001
Pseudo *R*^2^	0.28	0.33

### Sensitivity analysis

In unadjusted and adjusted analyses, at the population level, changes in the proportion of tobacco smokers who endorsed the belief that e-cigarettes are less harmful than combustible cigarettes (also categorising ‘do not know’ responses as endorsing the belief) were significantly associated with changes in the proportion who use e-cigarettes (see Additional file 2: Table S1). For every 1% decrease in the mean prevalence of tobacco smokers who endorsed this belief, the mean prevalence of e-cigarette use decreased by 0.48%.

### Analyses stratified by age, sex and social grade

#### Age

In unadjusted and adjusted analyses, at the population level, changes in the proportion of tobacco smokers who endorsed the belief that e-cigarettes are less harmful than combustible cigarettes were significantly associated with changes in the proportion who use e-cigarettes in those aged 25–64 years and those aged 65+ years, such that for every 1% decrease in the mean prevalence of this belief, the mean prevalence of e-cigarette use decreased by 0.37% and 0.22%, respectively. In those aged 16–24 years, however, there was no significant association between this belief and the use of e-cigarettes (see Table 2 and Additional file 2: Fig. S2).

**Table 2:** Estimated percentage point change in the mean prevalence of e-cigarette users among tobacco smokers who endorse the belief that e-cigarettes are less harmful than combustible cigarettes during the study period (2014–2019), based on autoregressive integrated moving average with exogenous input (ARIMAX) models stratified by age, sex and social grade

	**16–24 years**		**25–64 years**		**65+ years**	
	**Unadjusted** **(95% CI,*p*value)**	**Adjusted** **(95% CI,*p*value)**	**Unadjusted** **(95% CI,*p*value)**	**Adjusted** **(95% CI,*p*value)**	**Unadjusted** **(95% CI,*p*value)**	**Adjusted** **(95% CI,*p*value)**
**Percentage change per 1% change in the mean prevalence of the exposure**						
Use of e-cigarettes	0.12 (− 0.15–0.38), 0.39	0.13 (− 0.12–0.39), 0.31	0.42 (0.17–0.66), < 0.001	0.37 (0.13–0.61), < 0.01	0.22 (0.08–0.36), < 0.01	0.22 (0.07–0.37), < 0.01
Current cigarette smokers	–	− 0.14 (− 0.38–0.10), 0.25	–	0.01 (− 0.23–0.24), 0.96	–	− 0.03 (− 0.25–0.19), 0.78
Tried to quit in the past year	–	0.17 (− 0.08–0.43), 0.18	–	0.18 (− 0.07–0.42), 0.15	–	− 0.11 (− 0.34–0.13), 0.37
National mass media expenditure in millions (£)	–	− 0.11 (− 0.38–0.16), 0.43	–	0.16 (− 0.10–0.41), 0.23	–	0.08 (− 0.16–0.32), 0.50
**Best fitting model**						
ARIMAX (*p*,*d*,*q*)(*P*,*D*,*Q*)	(0,1,1)(0,0,0)	(0,1,1)(0,0,0)	(0,1,1)(0,0,0)	(0,1,1)(0,0,0)	(0,1,1)(0,0,0)	(0,1,1)(0,0,0)
Non-seasonal (*p* value)						
Autoregressive (AR) term	–	–	–	–	–	–
Moving average (MA) term	< 0.001	< 0.001	< 0.001	< 0.001	< 0.001	< 0.001
Pseudo *R*^2^	0.02	0.08	0.17	0.23	0.28	0.29
	**Men**		**Women**			
	**Unadjusted**	**Adjusted**	**Unadjusted**	**Adjusted**		
**Percentage change per 1% change in the mean prevalence of the exposure**						
Use of e-cigarettes	0.42 (0.18–0.66), < 0.001	0.48 (0.18–0.78), < 0.01	0.21 (− 0.07–0.49), 0.14	0.13 (− 0.12–0.38), 0.29		
Current cigarette smokers	–	− 0.21 (− 0.44–0.02), 0.08	–	0.13 (− 0.08–0.34), 0.23		
Tried to quit in the past year	–	0.18 (− 0.06–0.42), 0.15	–	0.37 (0.13–0.61), < 0.01		
National mass media expenditure in millions (£)	–	0.27 (− 0.08–0.62), 0.13	–	0.08 (− 0.17–0.33), 0.52		
**Best fitting model**						
ARIMAX (*p*,*d*,*q*)(*P*,*D*,*Q*)	(0,1,1)(0,0,0)	(0,1,1)(0,0,0)	(0,1,1)(0,0,0)	(0,1,1)(0,0,0)		
Non-seasonal (*p* value)						
Autoregressive (AR) term	–	–	–	–		
Moving average (MA) term	< 0.001	< 0.001	< 0.001	< 0.001		
Pseudo *R*^2^	0.23	0.25	0.16	0.31		
	**ABC1**		**C2DE**			
	**Unadjusted**	**Adjusted**	**Unadjusted**	**Adjusted**		
**Percentage change per 1% change in the mean prevalence of the exposure**						
Use of e-cigarettes	0.50 (0.28–0.72), < 0.001	0.49 (0.27–0.71), < 0.001	0.43 (0.18–0.67), < 0.001	0.37 (0.12–0.61), < 0.01		
Current cigarette smokers	–	0.06 (− 0.16–0.27), 0.59	–	− 0.05 (− 0.28–0.18), 0.68		
Tried to quit in the past year	–	0.06 (− 0.17–0.29), 0.61	–	0.28 (0.04–0.52), 0.02		
National mass media expenditure in millions (£)	–	0.15 (− 0.08–0.38), 0.21	–	0.07 (− 0.19–0.32), 0.60		
**Best fitting model**						
ARIMAX (*p*,*d*,*q*)(*P*,*D*,*Q*)	(0,1,1)(0,0,0)	(0,1,1)(0,0,0)	(0,1,1)(0,0,0)	(0,1,1)(0,0,0)		
Non-seasonal (*p* value)						
Autoregressive (AR) term	–	–	–	–		
Moving average (MA) term	< 0.001	< 0.001	< 0.001	< 0.001		
Pseudo *R*^2^	0.27	0.30	0.19	0.27		

#### Sex

In unadjusted and adjusted analyses, at the population level, changes in the proportion of tobacco smokers who endorsed the belief that e-cigarettes are less harmful than combustible cigarettes were significantly associated with changes in the proportion who use e-cigarettes in men, such that for every 1% decrease in the mean prevalence of this belief, the mean prevalence of e-cigarette use increased by 0.48%. For women, however, there was no significant association between this belief and the use of e-cigarettes (see Table 2 and Additional file 2: Fig. S3).

#### Social grade

In unadjusted and adjusted analyses, at the population level, changes in the proportion of tobacco smokers who endorsed the belief that e-cigarettes are less harmful than combustible cigarettes were significantly associated with changes in the proportion who use e-cigarettes in those with high and low social grade, such that for every 1% decrease in the mean prevalence of this belief, the mean prevalence of e-cigarette use decreased by 0.49% and 0.37%, respectively (see Table 2 and Additional file 2: Fig. S4).

## Discussion

### Principal findings

Between 2014 and 2019, at the population level, there was a decline in the proportion of tobacco smokers who endorsed the belief that e-cigarettes are less harmful than combustible cigarettes. There was also a decline in the proportion of tobacco smokers who reported the use of e-cigarettes during this time period. After adjusting for potential confounders and underlying trends, the decline in the belief among current smokers that e-cigarettes are less harmful than combustible cigarettes was strongly associated with declines in the use of e-cigarettes among current tobacco smokers in England. In analyses stratified by age, sex and social grade, some age and sex differences were observed, whereby significant associations were observed in older (but not in young) adults and in men (but not in women). No differences by social grade were detected.

The reduction in accurate harm perceptions is consistent with previously reported declining trends in the proportion of individuals who perceive e-cigarettes to be less harmful than conventional cigarettes in longitudinal surveys of nationally representative samples in the UK, Europe and the US [17, 20, 26, 27]. However, in contrast with the finding that the reduction in accurate harm perceptions was accompanied by an increase in ever use of e-cigarettes across several European countries [27], our results indicate that the reduction in accurate harm perceptions was accompanied by a decline in e-cigarette use among current tobacco smokers in England between 2014 and 2019.

### Strengths and limitations

To the authors’ knowledge, this was the first empirical study to estimate the association of the belief that e-cigarettes are less harmful than combustible cigarettes with e-cigarette use, using a time series approach. These results triangulate with previous studies that have used an individual-level approach to study the relationship between harm perceptions and e-cigarette use. Moreover, the population-level approach allowed us to take account of changes in the input and output series whilst adjusting for national mass media expenditure, which is known to influence smoking behaviour in England but cannot be sensibly incorporated into individual-level analyses [44]. This study was also strengthened by the use of a representative sample of current tobacco smokers in England.

This study had several limitations. First, although a total of 55 data points (or survey waves) were available for analysis, approximately 300 individual smokers were surveyed each month. The small sample size per survey wave might hence have influenced the precision of the estimates for the variables of interest. Although the sampling strategy used in the Smoking Toolkit Study is known to result in a sample that is representative of the general population of smokers in England with regard to demographic characteristics and cigarette consumption [31, 32], it is unclear whether the representativeness also applies to key psychological characteristics of smokers, such as beliefs and attitudes. Second, as there was no evidence for weak exogeneity between the input and output series, our results indicate that the association between harm perceptions and e-cigarette use was most likely one-directional, with harm perceptions influencing e-cigarette use, and not vice versa. It should, however, be noted that this association may be accounted for by a third, unmeasured variable or that some small bi-directionality may exist but that it was not detected in this study. Third, the Smoking Toolkit Study only asks current tobacco smokers about e-cigarette harm perceptions; it is hence unclear whether our findings generalise to past-year smokers or long-term ex-smokers. Fourth, the findings might not generalise to other countries: England has a strong tobacco control policy landscape and relatively liberal regulation of e-cigarettes; different relationships between e-cigarette harm perceptions and use may hence be observed in countries with weaker tobacco control policies or stricter regulation of e-cigarettes. Fifth, we had access to nominal (as opposed to real) national mass media expenditure. Hence, the numerical (real) value of a British pound in the first wave of 2014 may have differed from the last wave of 2019. Future research should endeavour to account for real expenditure. Sixth, although we adjusted for national smoking mass media expenditure, we were unable to take into account changes in e-cigarette media representations focusing on absolute (as opposed to relative) risks or exposure to e-cigarette marketing, which may have influenced both e-cigarette harm perceptions and use [11, 20, 24]. Seventh, similar to previous studies, we used a generic question to capture e-cigarette harm perceptions [17, 26, 27]. However, it is plausible that specific disease risk perceptions (e.g. about respiratory diseases or cancers) may differ from generic perceptions of harm and display different associations with e-cigarette use. Finally, this study assessed current e-cigarette use, but did not explore whether harm perceptions are differentially associated with the length, frequency or type of e-cigarette use. Future research should explore this further.

### Implications for policy and practice

The reduction in the proportion of tobacco smokers who perceive e-cigarettes to be less harmful than combustible cigarettes from 2014 to 2019 and the associated reduction in the use of e-cigarettes may reflect smokers’ concerns about the uncertainty about the long-term health effects of e-cigarettes. These concerns may have been amplified by frequent media reports focusing on the absolute (as opposed to relative) health risks of e-cigarettes or graphic, highly emotive depictions of e-cigarette explosions or e-cigarette or vaping product use-associated lung injury (EVALI) in the US. In line with Huang and colleagues’ call for an increase in the availability of accurate risk information about e-cigarettes in mainstream media [17], our results highlight the need for an increase in media portrayals and public health campaigns focusing on the reduced health harms by switching from combustible tobacco to e-cigarettes and a reduction in alarmist media coverage of events such as EVALI [2, 4, 45]. The observation of a significant association between harm perceptions and e-cigarette use in older but not younger adults may be reflective of cognitive biases that specifically affect young people (e.g. invulnerability bias, optimism), which may override harm perceptions [46]. It is unclear why a significant association was observed in men but not in women; future research is required to elucidate this.

### Avenues for future research

To examine associations with general use, we grouped current tobacco smokers who reported use of e-cigarettes to quit smoking, cut down, in situations where smoking is not allowed or for any other reason. However, assessing whether harm perceptions are differentially associated with e-cigarette use for different reasons may provide a more nuanced overview of the relationship between harm perceptions and e-cigarette use at the population level. This constitutes an important avenue for future research. Some researchers believe that inaccurate harm perceptions may drive smokers to maintain dual use of combustible tobacco and e-cigarettes as opposed to stopping smoking. Future research should therefore explore whether the observed decline in accurate harm perceptions is accompanied by a decline in smoking cessation.

## Conclusion

Between 2014 and 2019 in England, at the population level, declines in the prevalence of accurate harm perceptions among current tobacco smokers were associated with declines in the use of e-cigarettes.

## Supplementary information

**Additional file 1.** Detailed analysis plan.

**Additional file 2.** Supplementary tables and figures.

## Data Availability

Data and R code are available from the corresponding author on reasonable request. The study analysis plan is registered on the Open Science Framework at https://osf.io/ze5hf.
