# Supplementary material for: Association between changes in harm perceptions and e-cigarette use among current tobacco smokers in England: a time series analysis
Source: BMC Med. 2020 May 6;18:98. doi: 10.1186/s12916-020-01565-2 (PMC7201665; doi:10.1186/s12916-020-01565-2)
Supplement: Supplementary file 1 — Additional file 1. Detailed analysis plan. [file 12916_2020_1565_MOESM1_ESM.docx]

**Additional File 1**

The ARIMAX analyses proceeded in a number of stages:

1. Each time series was assessed for any outlying values which can bias the results using the *tsoutlier* package ^1^. This involved fitting a loess curve and identifying residuals as outliers if they were outside the range of:
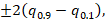
 where the
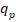
 is the p-quartile of the residuals. No outlying values were detected.
2. ARIMAX models require data to be stationary, meaning that the mean and variance of the time series are stable over time ^2^. Any observed trend in the output series is hence removed by replacing the value of the series at each point with the difference between that point and the value of the previous survey wave. A stationary time series fluctuates around a well-defined mean and the autocorrelation function (ACF) decays rapidly to zero (see Beard et al., 2019). Plots of the ACF and the partial autocorrelation function (PACF), and the Augmented Dickey-Fuller Unit Root Test were used to determine the number of seasonal and non-seasonal differences required for the time series to be made stationary ^4,5^. First-order differencing was sufficient for rendering the series stationary and no seasonal differencing was required. A natural log-transformation was used to stabilise the variance of the output series. To facilitate comparison across the main and stratified analyses, the time series were standardised by subtracting the mean, dividing by the standard deviation and adding a constant of 10 (to prevent negative values).
3. Plots of the ACF and PACF were used to identify plausible values for the AR and MA terms for the baseline model. Table 1 shows recommendations for identifying autocorrelation processes (adapted from <http://www.statsoft.com/Textbook/Time-Series-Analysis#arima>).
4. The cross-correlation functions were assessed with pre-whitened data to identify the most appropriate transfer functions (i.e. the manner in which past values of the input time series predict future values of the output time series) for the explanatory variables ^6^. Pre-whitening removes autocorrelation in the input time series, which may cause spurious cross-correlation effects.
5. ARIMAX models assume that there is weak exogeneity between the input and output time series, meaning that the output series can depend on lagged values of the input series but that the input series should not depend on lagged values of the output series ^2^. To assess this, we used the Granger Causality Test, which regresses each time series onto lagged values of itself and of the other time series ^2^. The Granger Causality Test suggested that there was evidence for the violation of the assumption of weak exogeneity between the input series (i.e. e-cigarette harm perceptions) and the output series (i.e. e-cigarette use) in the analysis stratified by age (i.e. in those aged 25-64 years). Caution has been advised when using this and similar tests on long time series ^7^, and there was no theoretical reason we could identify for a bidirectional relationship between e-cigarette harm perceptions and e-cigarette use only in those aged 25-64 years (and not in the main analysis). It was hence assumed that the association was spurious and likely removed following adjustment for other covariates.
6. Different models with various plausible AR and MA terms were then compared with the baseline model using the Akaike Information Criterion (AIC), with smaller values indicating better model fit. According to the Box-Jenkins method, in ARIMA (p, d, q) models, the total number of parameters should be less than 3 ^8^. Therefore, we only considered ARIMAX models with p and q values of 3 or less.
7. The Ljung-Box test for white noise ^9^ and the ACF for the residuals of the best fitting models were assessed for additional correlation (and hence the need for additional seasonal or non-seasonal AR or MA terms). The coefficients of the correlation terms were assessed for significance and whether they fell within the bounds of stationarity and invertibility ^2^, which means that recent, past observations are given more weight than distant, past observations.
8. Coefficients were reported for the best fitting models alongside the pseudo R^2^, calculated as the squared correlation of fitted to actual values. As differencing was used to render the time series stationary, the model coefficients can be interpreted as the percentage change in the mean of the output series (i.e. e-cigarette use) as a result of a 1% increase or decrease in the mean of the input series (i.e. e-cigarette harm perceptions).

**References**

1. Chen C, Liu L-M. Joint Estimation of Model Parameters and Outlier Effects in Time Series. *J Am Stat Assoc*. 1993;88(421):284-297.

2. Yaffee R. *An Introduction to Forecasting Time Series with Stata*. Taylor & Francis; 2012.

3. Beard E, Marsden J, Brown J, Tombor I, Stapleton J, Michie S. Understanding and using time series analyses in addiction research. *Addiction*. 2019:1-38. doi:10.1111/add.14643

4. Lee D, Schmidt P. On the power of the KPSS test of stationarity against fractionally-integrated alternatives. *J Econom*. 1996;73:285-302.

5. Osborn DR, Chu APL, Smith JP, Birchenhall CR. Seasonality and the order of integration for consumption*. *Oxf Bull Econ Stat*. 1988;50(4):361-377.

6. Cryer JD, Chan K-S. *Time Series Analysis: With Applications in R*. 2nd ed. New York: Springer-Verlag; 2008.

7. Granger CWJ. Some recent developments in a concept of causality. *J Econom*. 1988;39:199-211.

8. Box GEP, Jenkins GM, Reinsel GC. *Time Series Analysis: Forecasting and Control*. John Wiley & Sons, Inc.; 2011.

9. Ljung GM, Box GEP. On a measure of lack of fit in time series models. *Biometrika*. 1978;65(2):297-303. doi:10.1093/biomet/65.2.297
