# Supplementary material for: Association between changes in harm perceptions and e-cigarette use among current tobacco smokers in England: a time series analysis
Source: BMC Med. 2020 May 6;18:98. doi: 10.1186/s12916-020-01565-2 (PMC7201665; doi:10.1186/s12916-020-01565-2)
Supplement: Supplementary file 2 — Additional file 2. Supplementary tables and figures. [file 12916_2020_1565_MOESM2_ESM.docx]

**Additional File 2**

**Planned sensitivity analysis**

*Table S1.* Estimated percentage point change in the mean prevalence of e-cigarette users among respondents who endorse the belief that e-cigarettes are less harmful than combustible cigarettes (also categorising ‘don’t know’ responses as endorsing this belief) during the study period (2014-2019), based on autoregressive integrated moving average with exogenous input (ARIMAX) models.

|  | **Unadjusted**  **(95% CI, *p*-value)** | **Adjusted**  **(95% CI, *p*-value)** |
| --- | --- | --- |
| **Percentage change per 1% change in the mean prevalence of the exposure** |  |  |
| Use of e-cigarettes | 0.52 (0.30-0.75), <0.001 | 0.48 (0.24-0.71), <0.001 |
| Current cigarette smokers | - | 0.04 (-0.19-0.27), 0.76 |
| Tried to quit in the past year | - | 0.13 (-0.11-0.37), 0.28 |
| National mass media expenditure | - | 0.09 (-0.15-0.33), 0.45 |
| **Best fitting model** |  |  |
| ARIMAX (p,d,q)(P,D,Q) | (0,1,1)(0,0,0) | (0,1,1)(0,0,0) |
| Non-seasonal (*p*-value) |  |  |
| Autoregressive (AR) term | - | - |
| Moving average (MA) term | <0.001 | <0.001 |
| Pseudo R^2^ | 0.29 | 0.34 |

**Smokers’ harm perceptions over time, using the original 4-level coding**


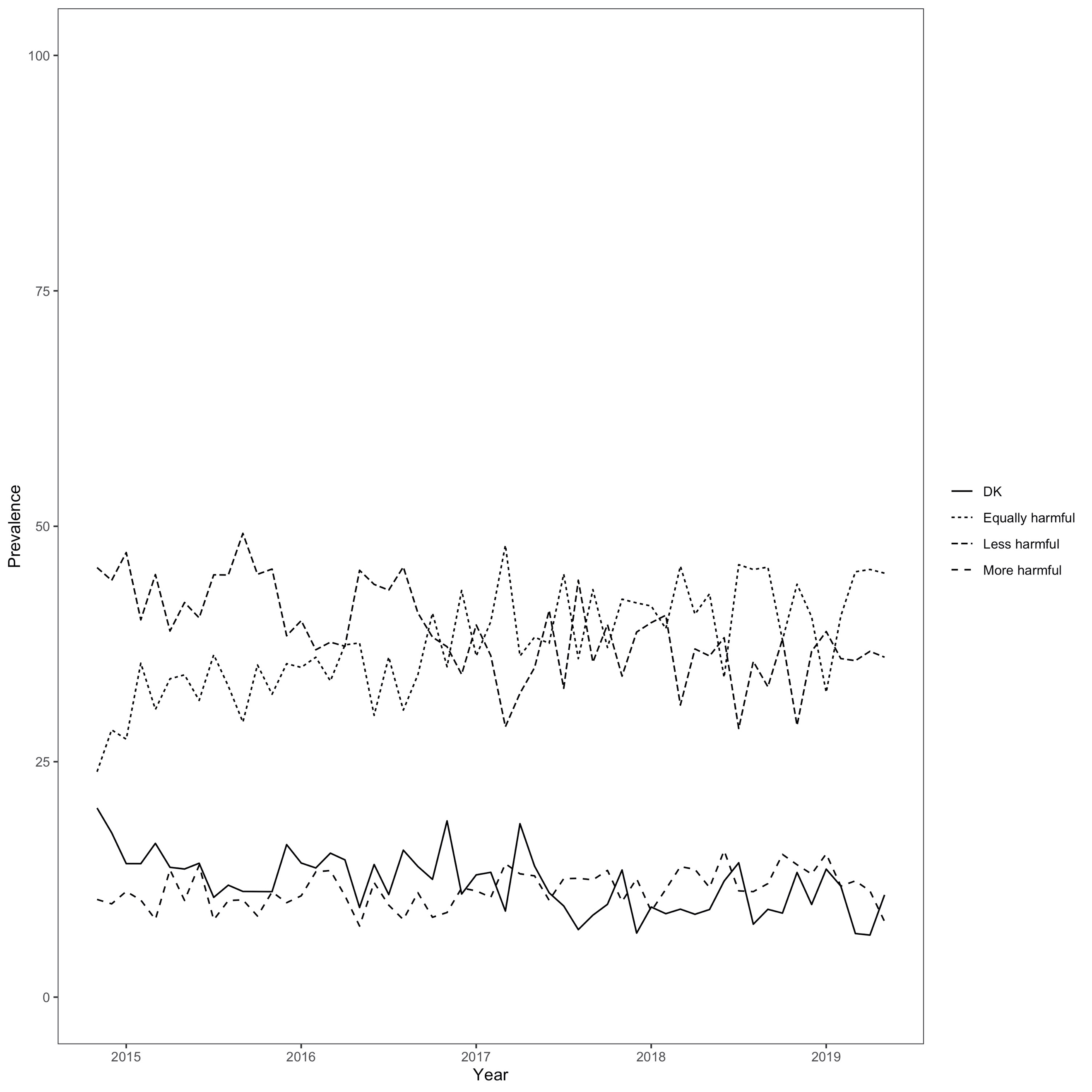

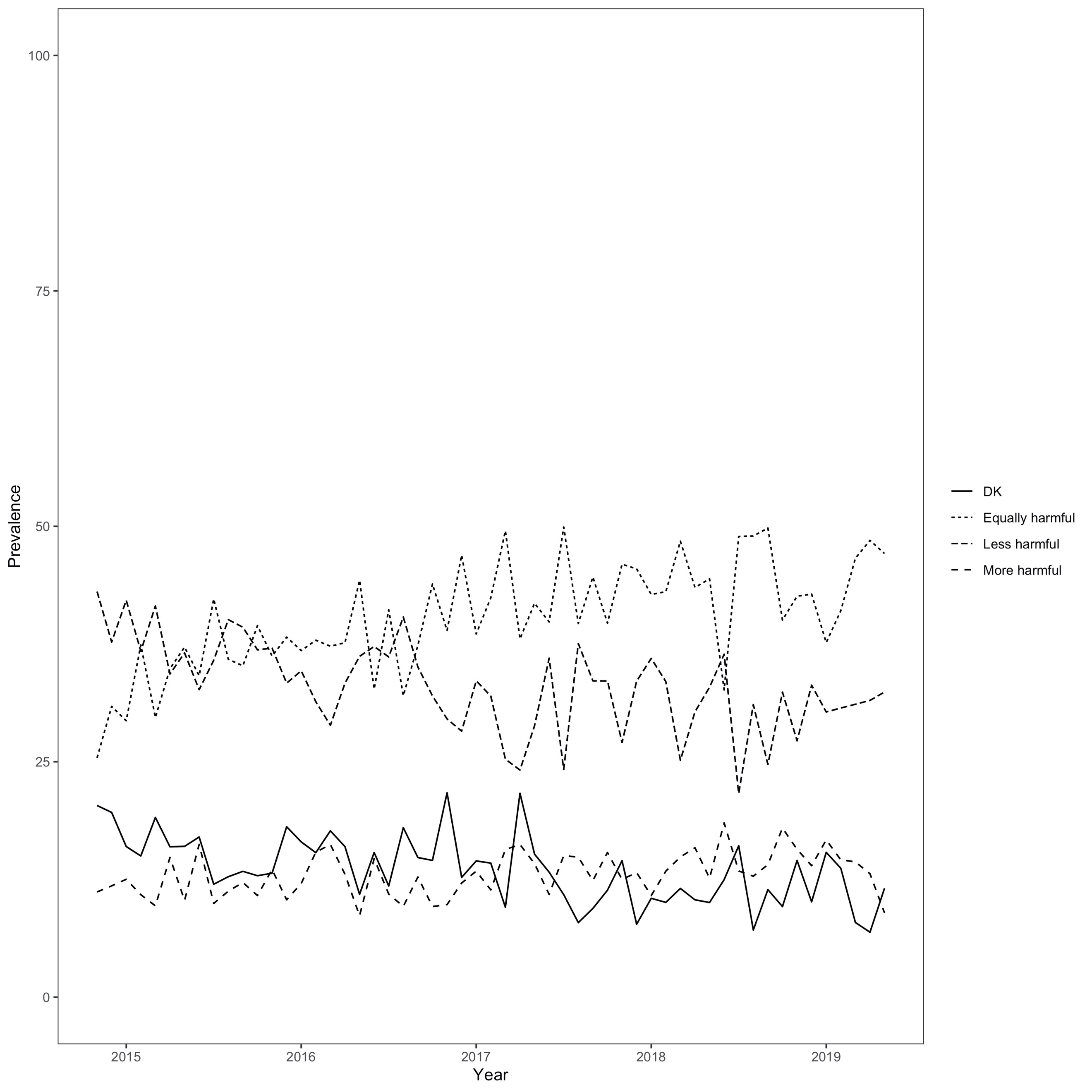


*Figure S1.* Descriptive plots of smokers’ harm perceptions over time in all current smokers (panel a) and current smokers who are not currently using e-cigarettes (panel b). DK = ‘don’t know’.

**Analyses stratified by age**


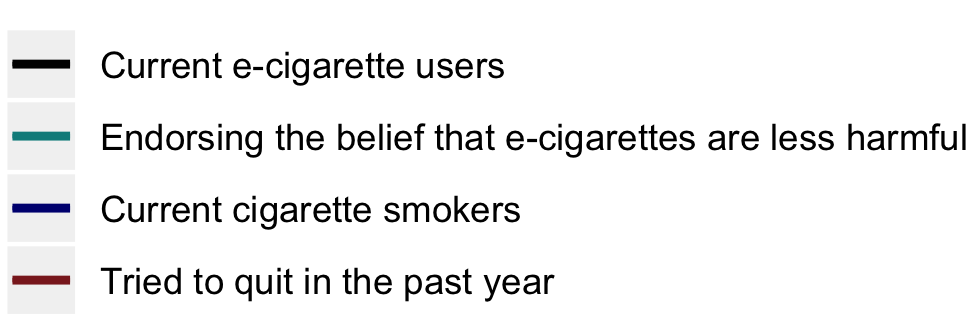


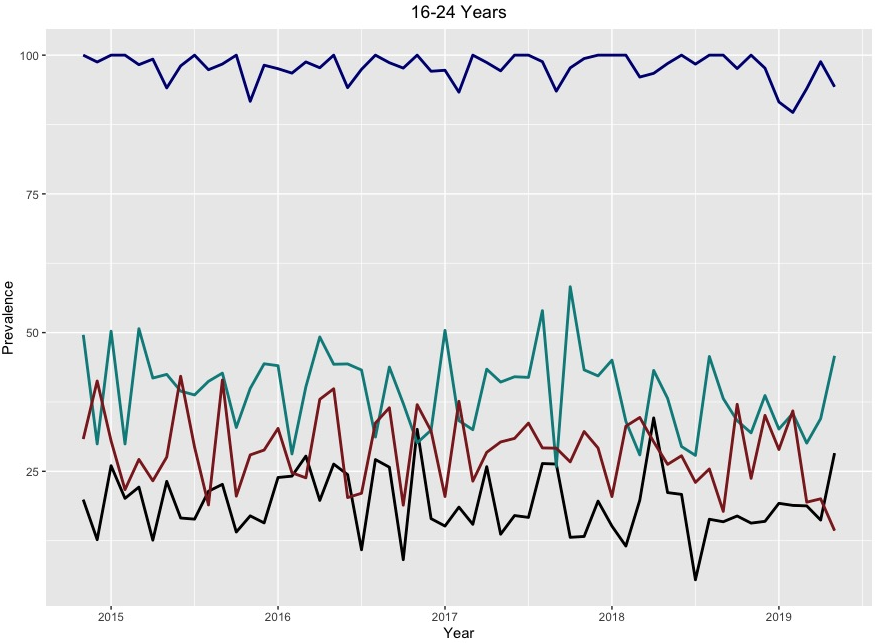

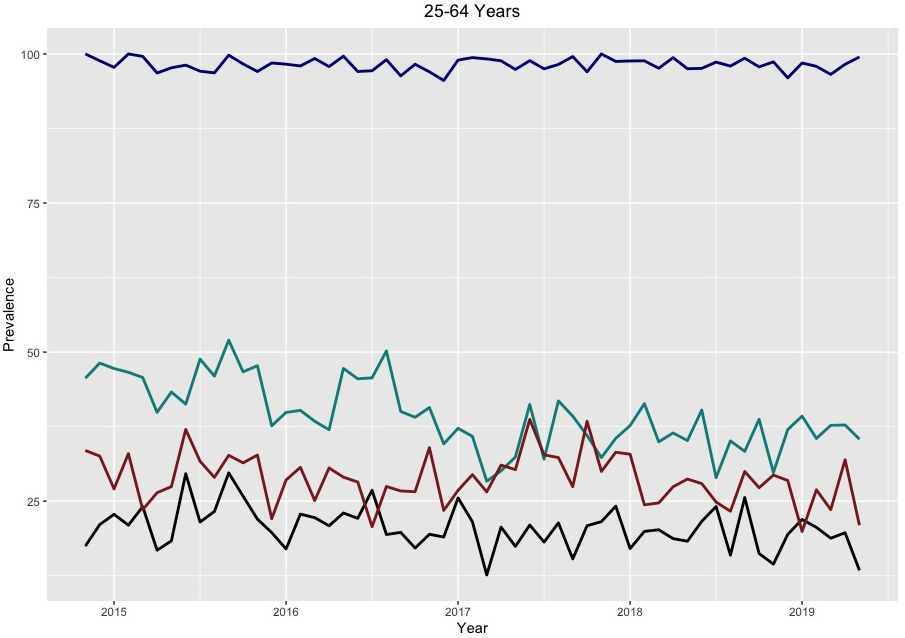


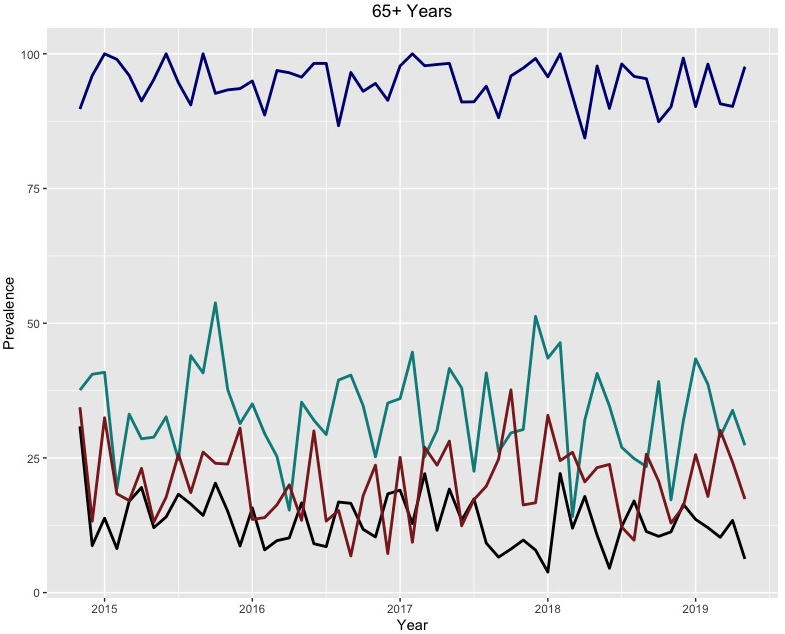


*Figure S2.* Monthly prevalence of e-cigarette users, cigarette smokers, respondents endorsing the belief that e-cigarettes are less harmful than combustible cigarettes and quit attempts in the past year in 16-24-year-olds (top left panel), 25-64-year-olds (top right panel) and respondents aged 65+ years (bottom panel).

**Analyses stratified by sex**


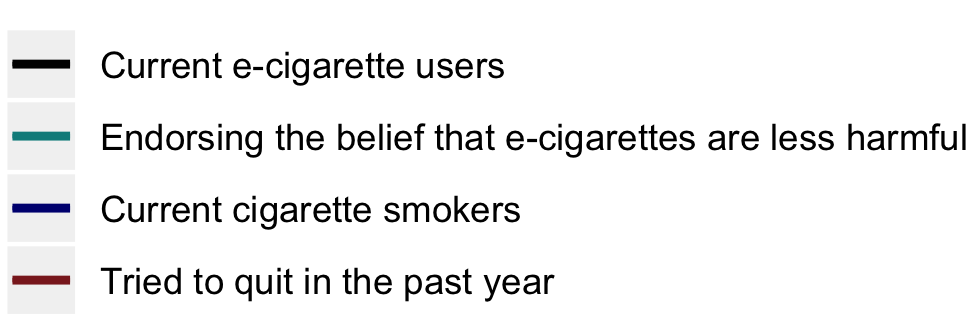


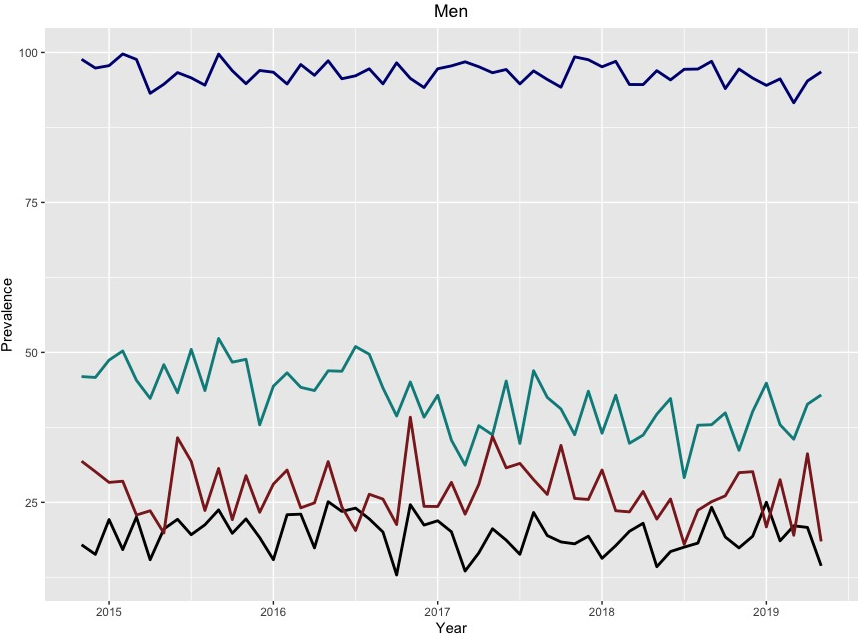

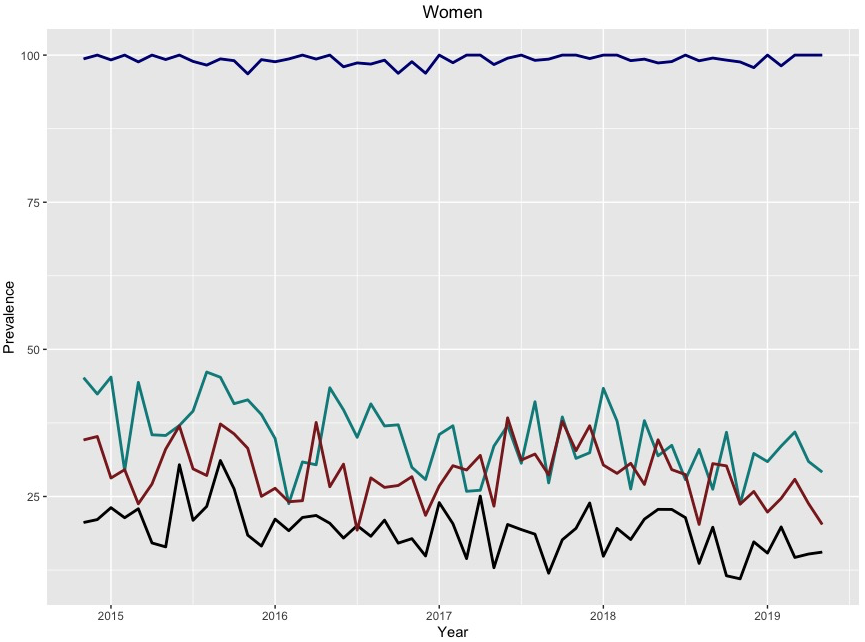


*Figure S3.* Monthly prevalence of e-cigarette users, cigarette smokers, respondents endorsing the belief that e-cigarettes are less harmful than combustible cigarettes and quit attempts in the past year in men (left panel) and women (right panel).

**Analyses stratified by social grade**


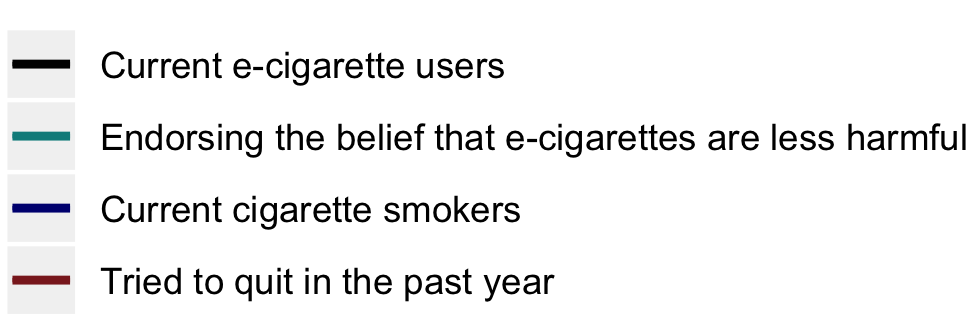


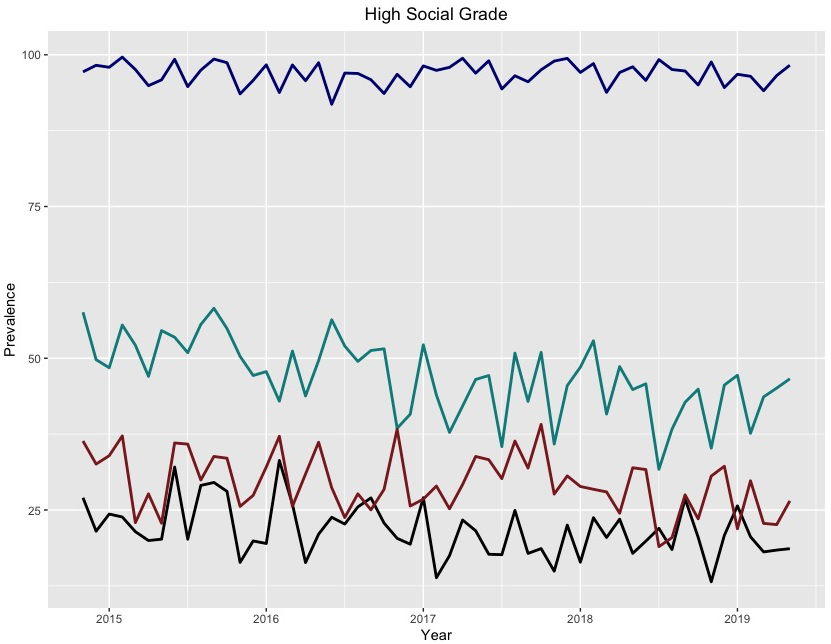

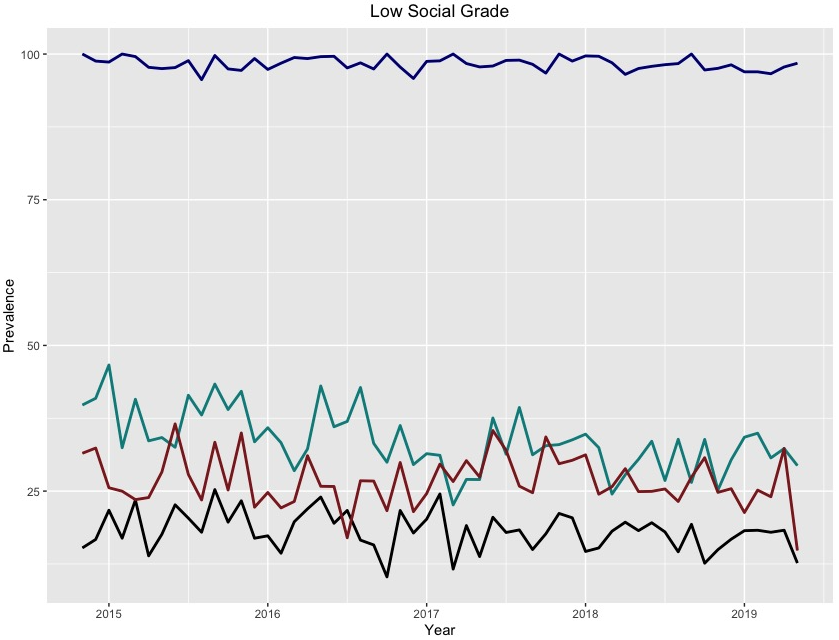


*Figure S4.* Monthly prevalence of e-cigarette users, cigarette smokers, respondents endorsing the belief that e-cigarettes are less harmful than combustible cigarettes and quit attempts in the past year in respondents with high (left panel) and low (right panel) social grade.

**Fitted compared with actual values of the output time series**

***
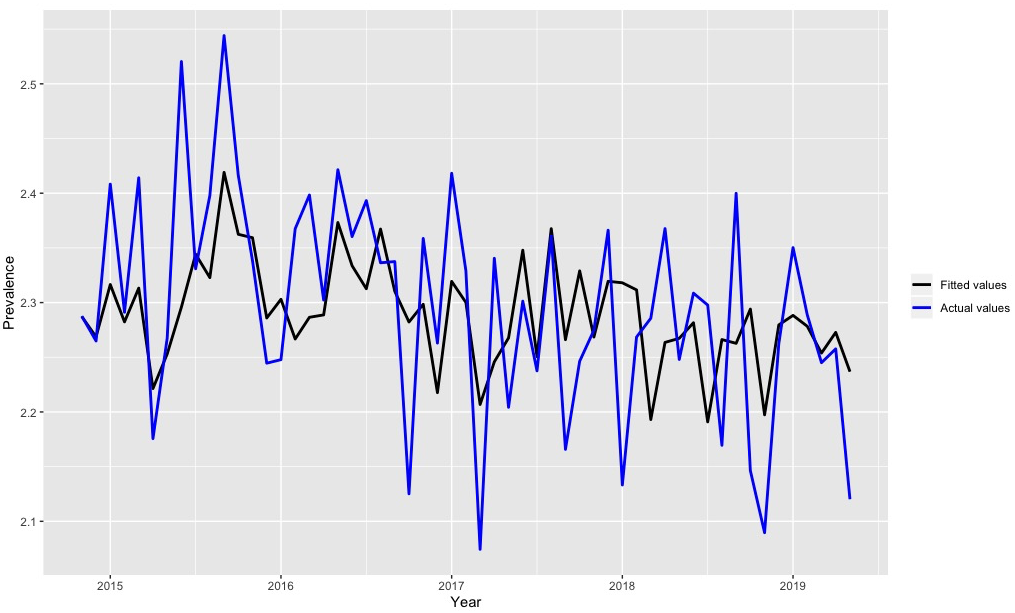
***

*Figure S5.* Association between harm perceptions and e-cigarette use in the total sample.

*
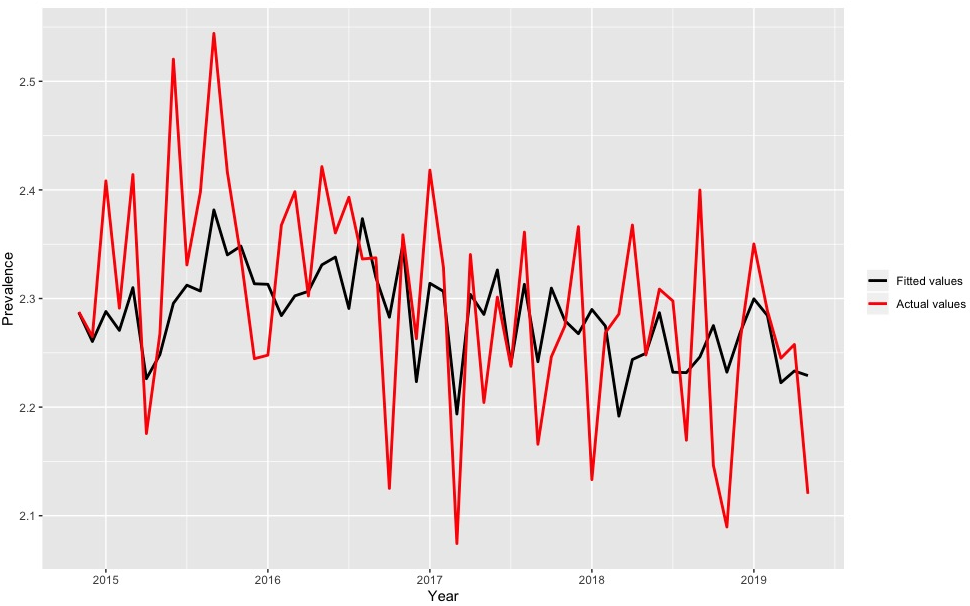
*

*Figure S6.* Planned sensitivity analysis.

*
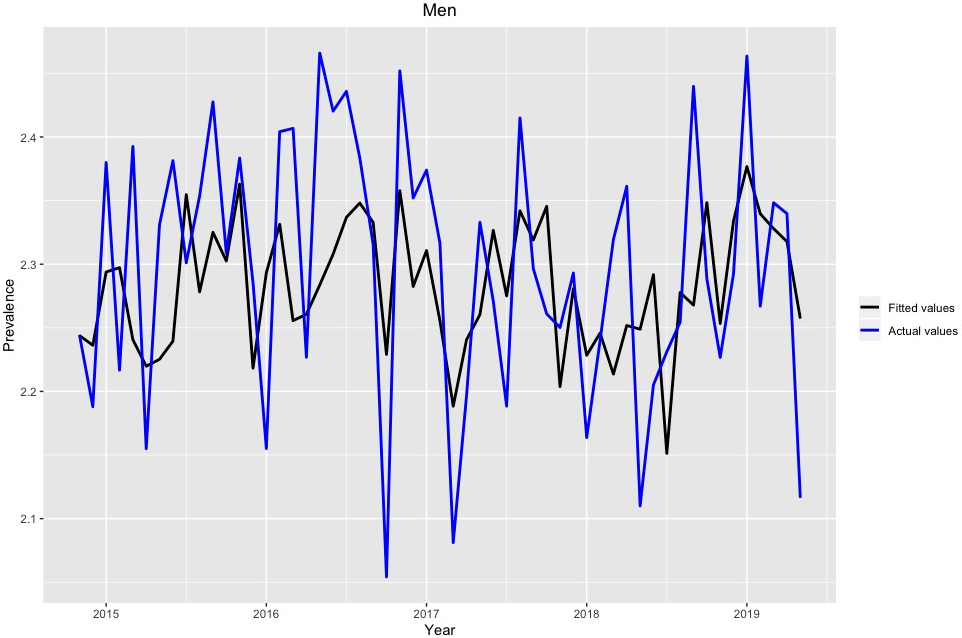

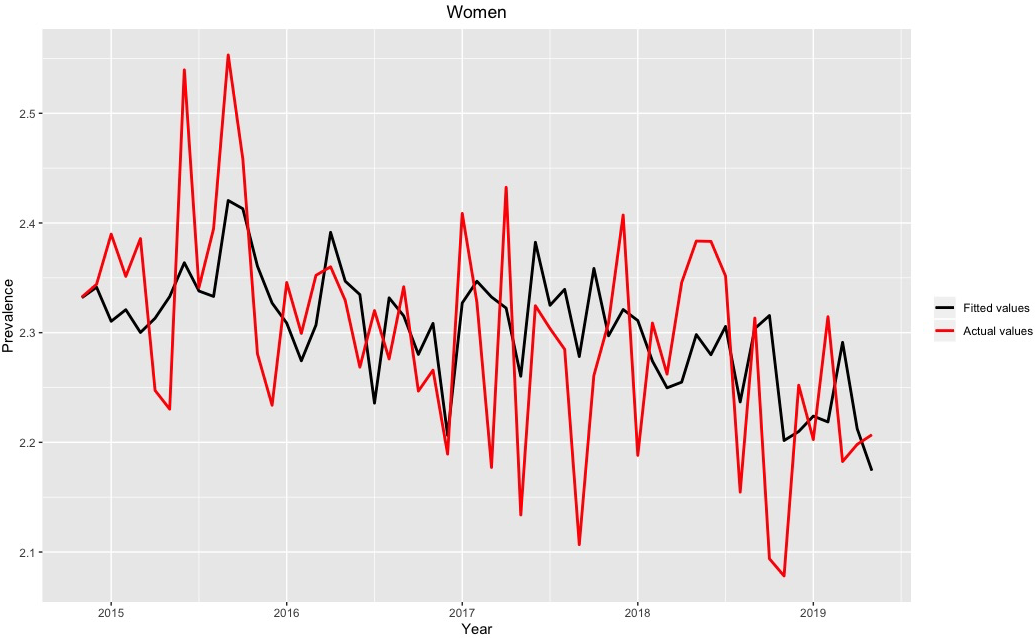
*

*Figure S7.* Analyses stratified by gender.


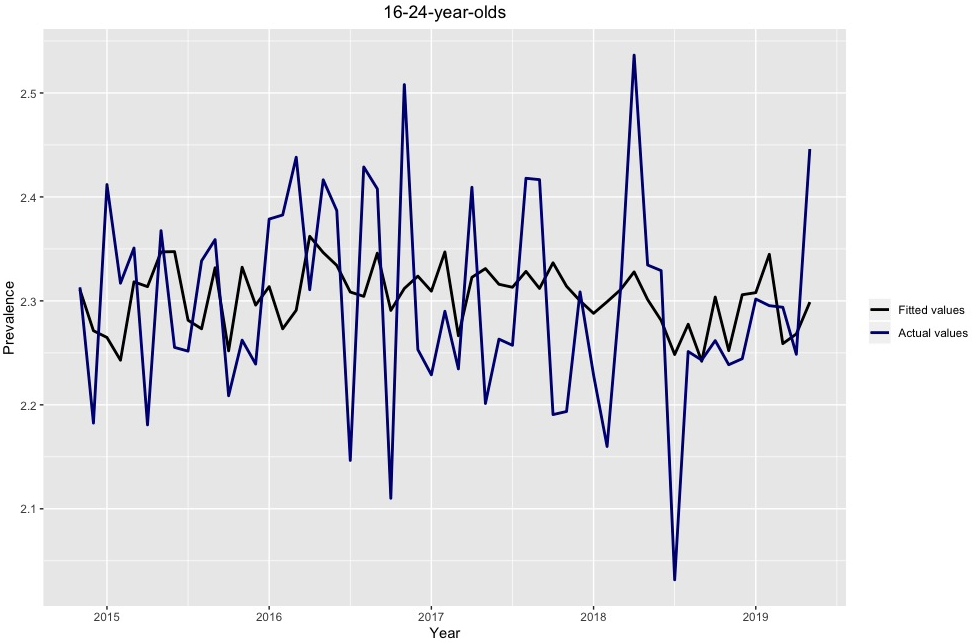

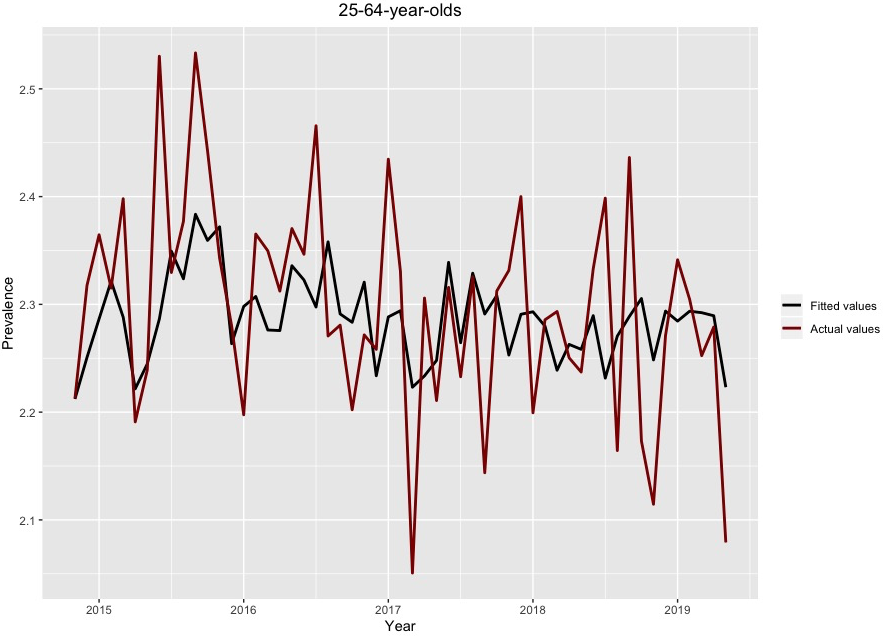


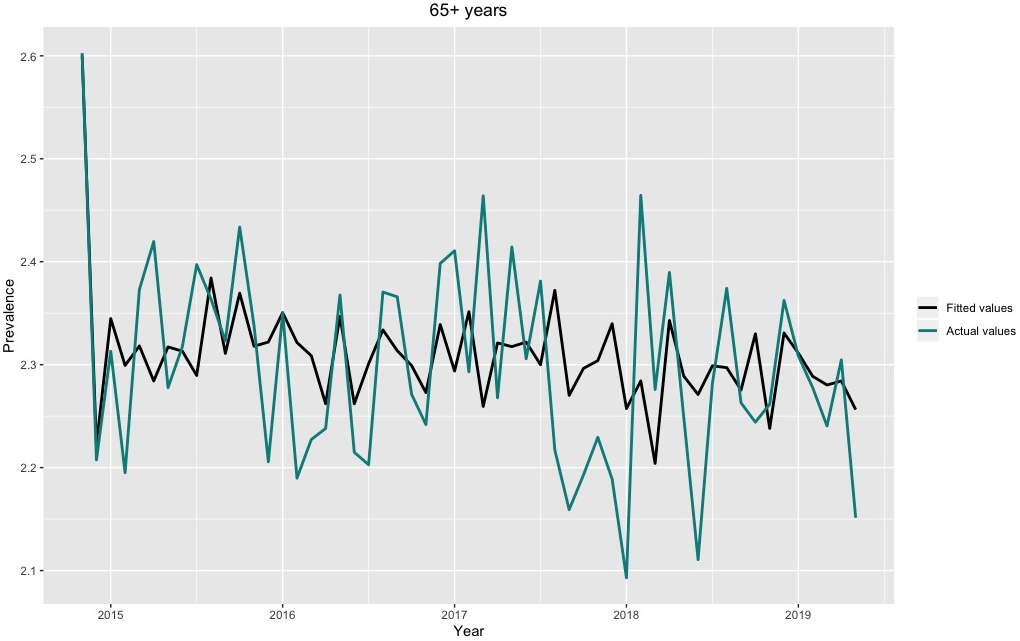


*Figure S8.* Analyses stratified by age.

*
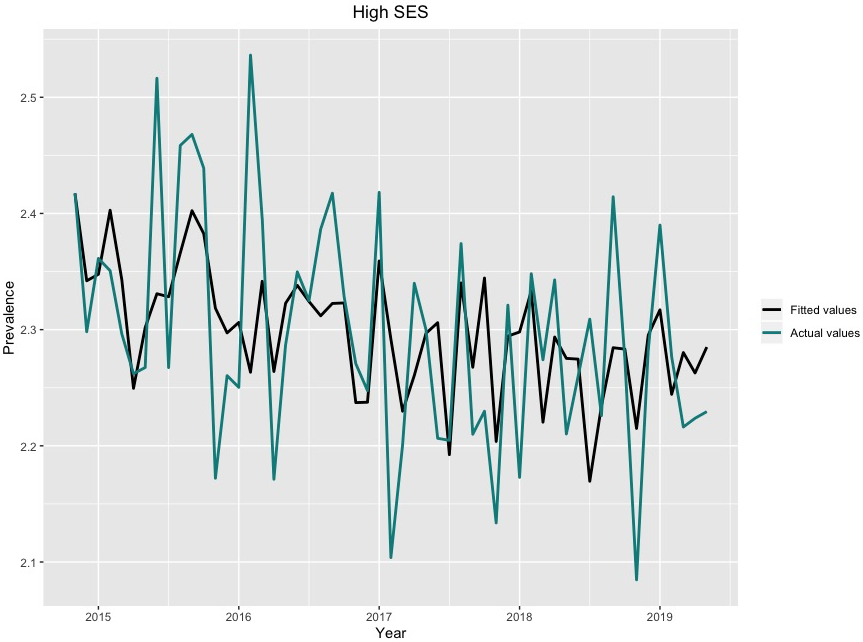

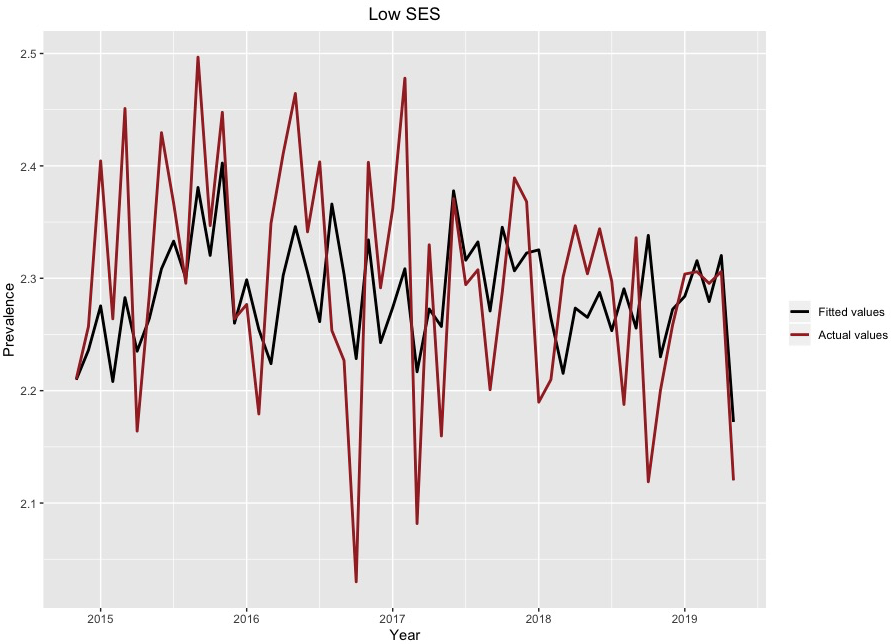
*

*Figure S9.* Analyses stratified by social grade.
